# Comprehensive Investigation of Areae Gastricae Pattern in Gastric Corpus using Magnifying Narrow Band Imaging Endoscopy in Patients with Chronic Atrophic Fundic Gastritis

**DOI:** 10.1111/j.1523-5378.2012.00938.x

**Published:** 2012-03-20

**Authors:** Hiromitsu Kanzaki, Noriya Uedo, Ryu Ishihara, Kengo Nagai, Fumi Matsui, Takashi Ohta, Masao Hanafusa, Noboru Hanaoka, Yoji Takeuchi, Koji Higashino, Hiroyasu Iishi, Yasuhiko Tomita, Masaharu Tatsuta, Kazuhide Yamamoto

**Affiliations:** * Department of Gastrointestinal Oncology, Osaka Medical Center for Cancer and Cardiovascular DiseasesHigashinari-ku, Osaka, Japan; † Department of Gastroenterology and Hepatology, Okayama University Graduate School of Medicine, Dentistry, and Pharmaceutical SciencesOkayama, Japan; ‡ Department of Pathology, Osaka Medical Center for Cancer and Cardiovascular DiseasesHigashinari-ku, Osaka, Japan

**Keywords:** Chronic atrophc fundic gastritis, image enhanced endoscopy, areae gastricae, magnified endoscopy, narrow band imaging

## Abstract

**Background:**

Barium radiographic studies have suggested the importance of evaluating areae gastricae pattern for the diagnosis of gastritis. Significance of endoscopic appearance of areae gastricae in the diagnosis of chronic atrophic fundic gastritis (CAFG) was investigated by image-enhanced endoscopy.

**Materials and Methods:**

Endoscopic images of the corpus lesser curvature were studied in 50 patients with CAFG. Extent of CAFG was evaluated with autofluorescence imaging endoscopy. The areae gastricae pattern was evaluated with 0.2% indigo carmine chromoendoscopy. Micro-mucosal structure was examined with magnifying chromoendoscopy and narrow band imaging.

**Results:**

In patients with small extent of CAFG, polygonal areae gastricae separated by a narrow intervening part of areae gastricae was observed, whereas in patients with wide extent of CAFG, the size of the areae gastricae decreased and the width of the intervening part of areae gastricae increased (*p* < 0.001). Most areae gastricae showed a foveola-type micro-mucosal structure (82.7%), while intervening part of areae gastricae had a groove-type structure (98.0%, *p* < 0.001). Groove-type mucosa had a higher grade of atrophy (*p* < 0.001) and intestinal metaplasia (*p* < 0.001) compared with foveola type.

**Conclusions:**

As extent of CAFG widened, multifocal groove-type mucosa that had high-grade atrophy and intestinal metaplasia developed among areae gastricae and increased along the intervening part of areae gastricae. Our observations facilitate our understanding of the development and progression of CAFG.

Chronic gastritis that is mainly caused by long-standing *Helicobacter pylori* infection leads to various gastric diseases including malignancy, and topography of chronic gastritis is closely related to different manifestations of gastric diseases; antral-predominant gastritis is seen in patients with duodenal ulcer, whereas corpus-predominant gastritis is frequently found in patients with gastric ulcer or gastric cancer [1]. Some studies have indicated a strong association between the endoscopically observed extent of chronic atrophic fundic gastritis (CAFG) and increased risk of development of gastric cancer in patients with *H. pylori* infection [2, 3].

The areae gastricae (AG) are defined as small, slightly elevated polygonal areas of the gastric mucosa 1–5 mm in diameter that was subdivided by the system of intersecting furrow on the mucosal surface and have been described in the standard textbook of histology for several decades [4]. Although importance of evaluating the AG pattern in making diagnosis of chronic gastritis was suggested in barium radiographic studies, significance of endoscopic appearance of the AG in the diagnosis of CAFG and their histological findings were not fully investigated [5]. Magnifying observation using image-enhanced endoscopy (IEE) such as chromoendoscopy or narrow band imaging (NBI) enables us to observe detailed morphological features of the mucosal surface of the digestive tract and increases the diagnostic yield of endoscopy to investigate pathogenesis of gastric diseases [6, 7]. Several studies have demonstrated that micro-mucosal structure observed by magnifying IEE represents the histological features of gastritis in patients with *H. pylori*-associated gastritis [8, 9].

The aim of this study, therefore, was to clarify the AG pattern in the gastric corpus and their micro-mucosal structures and histological finding by IEE and to speculate on their role in progression of CAFG in patients with *H. pylori*-associated gastritis.

## Methods

### Study Design and Setting

This was a cross-sectional study that was conducted in an endoscopy unit of a referral cancer center from February to December 2010. The study protocol was approved by the ethical committee in our institution.

### Participants

We enrolled patients with early-stage noncardiac gastric cancer who presented to undergo endoscopic submucosal dissection (ESD) and gave written informed consent to participate. The following were excluded: patients who had a history of *H. pylori* eradication therapy or gastric surgery, those with severe organ failure or bleeding tendency, and those who were receiving anticoagulant, antiplatelet, or nonsteroidal anti-inflammatory drugs within 7 days of the study.

### Endoscopic Procedures

All endoscopic evaluation was performed before ESD procedure under sedation using a trimodal imaging endoscopy system that consisted of a light source (CLV-260SL; Olympus Medical Systems, Tokyo, Japan), a processor (CV-260SL; Olympus), a video monitor, and a magnifying videoendoscope (EVIS-FQ260Z; Olympus) that was equipped with two charged coupled devices: one for high-definition white-light imaging (WLI) and NBI modes with zoom function and another for autofluorescence imaging (AFI). A black rubber cap (MB-46; Olympus) was fitted to the tip of the videoendoscope to maintain adequate distance from the mucosa for magnifying observation. Before starting intubation, the black cap was fitted deeply enough in each WLI and AFI mode until only the tip of the cap could be seen because the cap could partially disrupt the imaging field. Structural enhancement level was set at B4 or B6 in WLI, B8 in NBI, and A3 in AFI modes.

The patients ingested a mixture consisting of a mucolytic agent (20,000 U pronase, Pronase MS; Kaken Pharmaceutical, Tokyo, Japan), a defoaming agent (80 mg dimethylpolysiloxane syrup, Gascon Drops; Kissei Pharmaceutical, Matsumoto, Japan), and 1 g sodium bicarbonate diluted in 100 mL tap water, 5 min before the examination. After topical anesthesia, the endoscope was gently inserted into the stomach. When food residue, mucous, or bubbles were on the mucosal surface, they were washed with 60–100 mL 0.04% dimethylpolysiloxane solution (Kissei Pharmaceutical) before observation. At first, the extent of CAFG was evaluated with AFI mode and at least two images of each downward and retroflex views of the gastric corpus were recorded. For evaluation, the lumen of the gastric body was adequately distended with sufficient air insufflation to obtain good images. After that, 15 mL 0.2% Indigo carmine (Daiichi Sankyo, Tokyo, Japan) solution that was filled in a 20-mL disposable syringe was administered onto the mucosal surface through the working channel. Indigo carmine was administered to the greater curvature of the antrum first and then flushed to the lesser curvature of the corpus. Subsequently, air was suctioned and the gastric lumen collapsed to apply the dye solution to the whole gastric mucosa. After repeated air inflation, the AG pattern in the lesser curvature of the corpus was evaluated. Finally, micro-mucosal structure of the lesser curvature of the lower corpus was assessed by magnifying endoscopy with chromoendoscopy and NBI. The region-of-interest to investigate the AG and micro-mucosal patterns measured 2 × 2 cm at the corpus lesser curvature, 4 cm proximal to the gastric angulus. One region-of-interest was evaluated in each patient. The reason why we decided on this region-of-interest for assessing micro-mucosal structure pattern was based on the hypothesis that CAFG usually develops in the corpus lesser curvature and subsequently progresses upwards to the cardia and laterally to the anterior and posterior wall side [10]. We assumed, therefore, that the area in the corpus lesser curvature represented early-stage mucosal appearance of CAFG in patients with narrow CAFG, whereas the area represented late-stage mucosal appearance of CAFG in patients with extensive CAFG. Target biopsies were taken from different micro-mucosal structures under magnifying observation. If the region-of-interest consisted of the same micro-mucosal structure, one biopsy specimen was taken from the center of the area.

### Extent of CAFG

**Figure 1:**
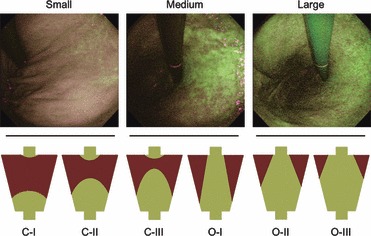
Extent of chronic atrophic fundic gastritis in autofluorescence imaging. A small area of green mucosa was observed only in the lower part of the corpus lesser curvature (small), whereas the green mucosa extended upward to the cardia and the color border was seen in the lesser curvature (medium). The green mucosa distributed to the anterior and posterior wall, and almost all the area was seen as green mucosa (large). Corresponding illustrations of Kimura-Takemoto classification (C-I to O-III) were exhibited below.

The extent of CAFG was examined in AFI mode and was classified into six categories according to the Kimura–Takemoto classification as follows [11,12]: C-I, the entire gastric body looked purple to dark green; C-II, a color border on the lesser curvature was observed at a lower part of the gastric body; C-III, a color border on the lesser curvature was observed at an upper part of the gastric body; O-I, a color border was observed between the lesser curvature and the anterior wall; O-II, a color border was observed between the anterior wall and the greater curvature; and O-III, a color border on the greater curvature was observed proximal to the lower gastric body. The CAFG was regarded as “small” for C-I and C-II, “medium” for C-III and O-I, and “large” for O-II and O-III (Fig. 1).

### AG Pattern

**Figure 2:**
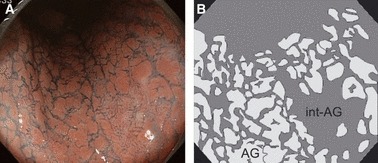
Areae gastricae (AG) pattern in chromoendoscopy. Polygonal AG that looked reddish in chromoendoscopy were arranged in the corpus lessor curvature, and there was an int-AG that appeared bluish (*A*); the AG were indicated as light gray and the int-AG was indicated as dark gray in schematic images (*B*).

Although AG is basically a term in anatomy and double-contrast barium radiography [13], they were observed as flat or slightly protruded polygonal areas of mucosa that were divided by interconnecting sulci gastricae that retained indigo carmine on chromoendoscopic imaging [5]. When we evaluated the appearance of AG in patients with *H. pylori*-associated gastritis in our early study [14], we noticed that AG were often divided by wide areas, especially in the gastric corpus. Therefore, we defined these areas that existed in between AG as the intervening part of AG (int-AG) (Fig. 2). The AG pattern was classified into the following categories according to the proportion of AG and int-AG in the region-of-interest: AG type: almost all (>80%) areas consisted of AG divided by the sulci gastricae or thin int-AG; AG>int-AG type, AG occupied 80–50%; AG<int-AG type, int-AG occupied 80–50%; and int-AG type, almost all (>80%) areas were occupied by int-AG and little AG were seen.

### Micro-Mucosal Structure Pattern

**Figure 3:**
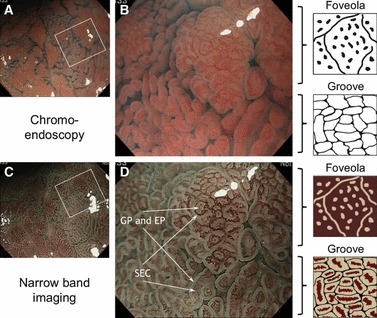
Micro-mucosal structure pattern in magnifying chromoendoscopy and narrow band imaging (NBI). White squares in (*A*) and (*C*) indicate the magnified area taken for images in (*B*) and (*D*), respectively. The foveola type was characterized as mucosa with round, oval, or linear foveolae (gastric pits) in chromoendoscopic images, whereas the groove type was characterized by mucosal crests that were divided by continuous grooves. On NBI, the gastric pits and adjacent epithelia appear as light brown areas, and these were surrounded by dark brown subepithelial capillaries in the foveola type. The dark brown subepithelial capillaries were surrounded by light brown gastric pits and epithelium in the groove type. GP, gastric pit; EP, epithelium; SEC, subepithelial capillary.

Under observation at the maximum magnification, micro-mucosal structure in the AG and the int-AG was evaluated. Micro-mucosal structure was classified into two types: foveola and groove, according to magnifying chromoendoscopic [16] and NBI [17] findings. The foveola type was characterized as mucosa with round, oval, or linear foveolae (gastric pits) in magnifying chromoendoscopic images. In magnifying NBI, the foveola-type mucosa has dark brown areas that surround light brown areas. The groove type was characterized by mucosal crests that were divided by continuous grooves on chromoendoscopic image and, on NBI, mucosa with light brown areas that surrounds dark brown areas (Fig. 3). In NBI image, a dark brown area corresponds to the subepithelial capillary. The subepithelial capillary sometimes looks like a single dilated vessel, whereas it sometimes appears as a brownish belt of twined fine vessels. A light brown area corresponds with the gastric pits and the epithelium.

### Histological Assessment

All of the biopsy specimens were stained with hematoxylin and eosin. Mononuclear cell infiltration (inflammation), neutrophil infiltration (activity), glandular atrophy (atrophy), and intestinal metaplasia were estimated and graded as normal, mild, moderate, or marked according to the visual analog scale in the updated Sydney system [18]. The histological findings were evaluated by one pathologist who was unaware of the endoscopic findings.

### Definition of *H. pylori* Status

At least two of the following were carried out in all patients: bacterial culture, serological *H. pylori* antibody test, or histological examination. We defined patients who are positive for any of *H. pylori* tests as “current infection.” Patients who are negative for all *H. pylori* tests but having intestinal metaplasia in the antrum or the corpus were regarded as “previous or latent infection” because progression of intestinal metaplasia in the gastric mucosa could cause spontaneous eradication of *H. pylori* or underestimation of *H. pylori* infection [19, 20]. Patients with neither positive result of *H. pylori* test nor intestinal metaplasia were defined as “not infected.”

### Study Size and Statistical Analysis

Sample size was determined according to the feasibility of sample collection and was set at 50 patients in the early part of the study. All statistical analyses were performed with computer statistical software (SPSS version 11.0; Chicago, IL). The relationship with the extent of CAFG and AG pattern was analyzed with Spearman’s rank correlation coefficient. Difference in micro-mucosal pattern between AG and int-AG was compared using the chi-square test with Yate’s correction. Grade of histological gastritis between different types of micro-mucosal pattern was compared by using the Wilcoxon signed rank test. A *p* value of <0.05 was considered to be statistically significant.

## Results

We studied 50 patients with early gastric cancer, including 44 men and six women with a median age of 69 years. Patients’ demographic details are listed in Table 1. The extent of CAFG in the study sample was classified as small in seven patients, medium in 15, and large in 28.

**Table 1:** Characteristics of patients is included this study

No. of patients	50
Median age (range, years old)	69 (45–81)
Sex (men/women)	44/6
*Helicobacter pylori* status (%)	
Current infection	28 (56)
Previous or latent infection	22 (44)
Not infected	0 (0)
Extent of atrophic fundic gastritis (%)	
Small	7 (14)
Medium	15 (30)
Large	28 (56)
Location of tumor	
Upper third	5 (10)
Middle third	8 (16)
Lower third	37 (74)
Histology of tumor	
Intestinal type	45 (90)
Undifferentiated type	5 (10)

### Association Between the Extent of CAFG and AG Pattern

**Figure 4:**
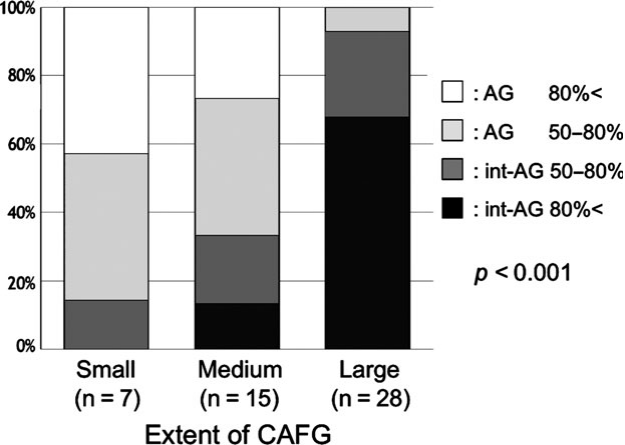
Association between the extent of chronic atrophic fundic gastritis and areae gastricae pattern.

In more than 80% of patients with small extent of CAFG, the region-of-interest basically consisted of AG that were divided by sulci gastricae or narrow int-AG. As the extent of CAFG increased, the int-AG also increased and >90% of patients with wide CAFG had an int-AG-dominant pattern with almost the entire area occupied by int-AG, and only a small AG remained in 50% of patients with large extent of CAFG (Fig. 4). There was a significant association between the extent of CAFG and AG pattern (*p* < 0.001).

### Association Between AG Pattern and Micro-Surface Pattern

**Figure 5:**
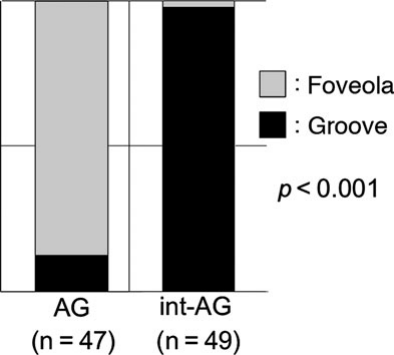
Association between areae gastricae and micro-mucosal structure.

One patient had only AG and three had only int-AG in the region-of-interest. The micro-mucosal pattern in the AG was foveola type in 87% (41/47) and that in the int-AG was groove type in 98% (48/49) (*p* < 0.001; Fig. 5).

### Histological Findings of Each Micro-Mucosal Pattern

**Figure 6:**
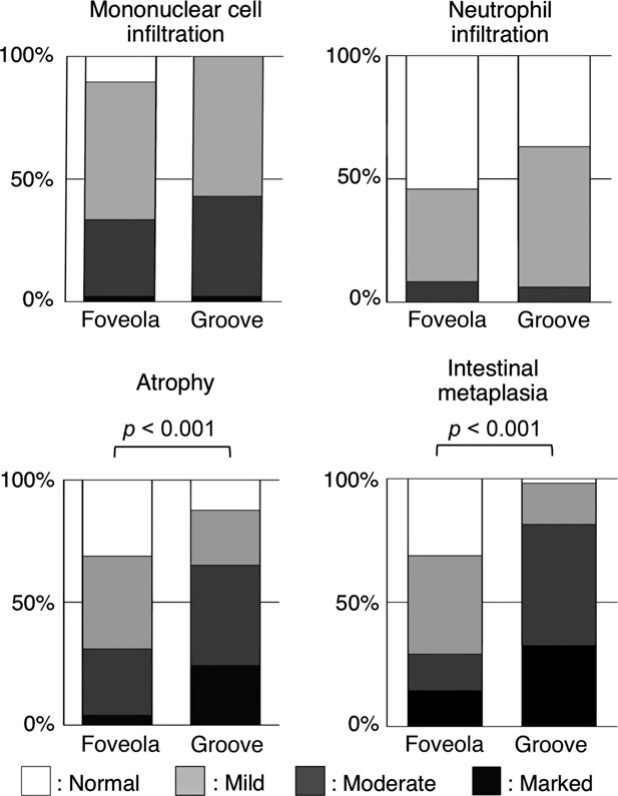
Association between micro-mucosal structure and grade of gastritis.

**Figure 7:**
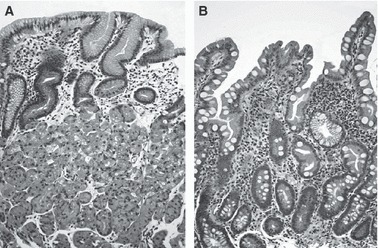
Histological finding of biopsy specimen taken from adjacent mucosa with different micro-mucosal structure (H&E, ×200). The foveola-type mucosa conserved oxyntic gland and had narrow straight pits (*A*). In the groove-type mucosa, oxyntic gland was lost and showed intestinal metaplasia. Wide gastric pits existed between papillary-shaped surfaced epithelia (*B*).

In 46 patients that had both foveola and groove patterns, two biopsy specimens were taken from each type of micro-mucosal pattern. One biopsy specimen was taken from one patient with only foveola type and from three patients with only groove type in the region-of-interest. Thus, a total of 96 biopsy specimens were evaluated. The grade of mononuclear cell and neutrophil infiltration was not significantly different between foveola and groove type. The grade of atrophy and intestinal metaplasia was significantly higher (both *p* < 0.001) in groove type compared with foveola type (Fig. 6). Most of the foveola type preserved oxyntic gland and was covered with the epithelium with narrow straight pits, whereas, in the Groove type, oxyntic gland decreased owing to atrophic/metaplastic change and the surfaces epithelium had wide gastric pits between papillary intervening epithelia (Fig. 7).

## Discussion

In this study, we revealed with IEE that there was a clear association between the extent of CAFG and the AG pattern in patients with chronic gastritis. As the extent of CAFG widened, the size of the AG decreased, compared with a proportional increase in int-AG at the corpus lesser curvature. The AG had foveola-type micro-mucosal structure and low-grade atrophy and intestinal metaplasia, whereas int-AG had groove-type micro-mucosal structure with high-grade atrophy and intestinal metaplasia.

In radiographic studies, AG had been demonstrated by graded compression or double-contrast method with careful barium examination, and altered AG pattern has been shown to be a good indicator of chronic gastritis [21]. However, some investigators have argued that the AG are not visualized when there is a thick mucous coat on the mucosal surface [22] and the AG pattern do not always represent underlying chronic gastritis [23]. Moreover, difficulty of direct comparison of radiographic appearance with histological finding and variation in histological criteria of gastritis had made significance of the AG patterns unknown to pathologists as well as radiologists. In the present study, there was a clear correlation between the extent of CAFG, AG pattern, micro-mucosal structure, and histology of gastritis. Preparation with a mucolytic agent to contrast the AG, targeted biopsy using zoom endoscopy, and the use of established histological criteria (updated Sydney system) enabled us to link gross endoscopic appearance to microscopic features.

Magnifying NBI revealed that most AG had a foveola-type micro-mucosal structure and int-AG had groove type. Although many investigators have proposed miscellaneous classifications that have multiple, sometimes four or more, categories to explain the micro-mucosal structure of the gastric mucosa in white light endoscopy [16,24,25], chromoendoscopy [26], and NBI [8,9] with magnification, we found that it could be classified into two major types: foveola and groove types. These categories were based on whether subepithelial capillaries that looked brownish with NBI encircled the gastric foveolar pit or not. Bansal et al. [17] have suggested this to explain the difference in micro-mucosal structures of normal mucosa in the corpus and antrum. They have demonstrated that, in gastric corpus mucosa, the capillary loops surround the gastric pits, whereas in the antral mucosa, subepithelial capillaries are situated in the micro-crest of the gastric epithelium. On NBI, therefore, dark brownish areas surround the light brownish areas in the corpus, whereas the light areas surround the dark areas in the antrum. Accordingly, in the corpus, development of the groove-type mucosa may represent metaplastic change of the oxyntic mucosa to mucosa mimicking pyloric (i.e., pseudo-pylorization) or intestinal (i.e., intestinalization) mucosa. Supporting this speculation, the groove-type mucosa often has a white–bluish fringe of light around the surface of the mucosal crests (light blue crest) in NBI images, which are a specific sign of intestinal metaplasia [27], whereas this is not observed in the foveola-type mucosa. Our two categories of micro-mucosal structures corresponded well with AG pattern and histology of chronic gastritis. Therefore, recognition of only these two micro-mucosal patterns could facilitate our understanding of the development and progression of CAFG in magnifying endoscopic imaging.

**Figure 8:**
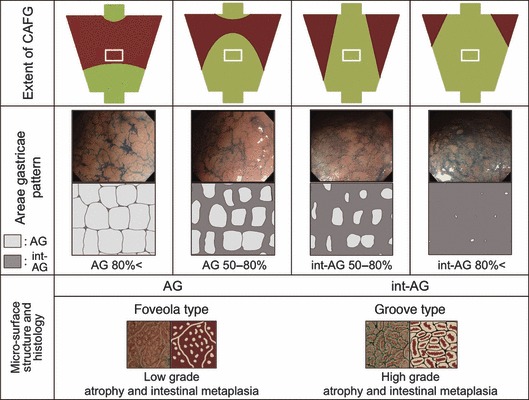
Suggested progression pattern of chronic atrophic fundic gastritis (CAFG) in image-enhanced endoscopy. As the extent of CAFG widened, int-AG increased, instead of a reduction in areae gastricae (AG). AG had foveola-type micro-mucosal structure with low-grade atrophy and intestinal metaplasia, whereas int-AG showed groove type with high-grade atrophy and intestinal metaplasia. A white square in the scheme of autofluorescence imaging indicated a region-of-interest for the micro-mucosal evaluation. AG, areae gastricae.

Based on pathological investigations, Correa et al. [28] have indicated that patchy areas of atrophic–metaplastic changes in the antral or corpus mucosa (i.e., multifocal atrophic gastritis) frequently coexist with gastric ulcers or gastric cancer. By autopsy, they have inferred from the pattern of progression of multifocal atrophic gastritis with age that focal atrophic–metaplastic mucosa first develops at the incisura angularis. New atrophic foci then appear along the lesser curvature in the antrum and corpus, and later, in the anterior and posterior walls. These foci coalesce with each other, resulting in extensive areas of atrophy that eventually may involve most of the gastric mucosa [29]. According to our observations, suggestive progression of CAFG at the corpus lesser curvature involved focal development of groove-type mucosa that had high-grade atrophy and intestinal metaplasia among AG; extension of the groove-type mucosa along the sulci gastricae connecting each other and forming int-AG; increased width of groove-type mucosa in int-AG encroaching upon foveola-type mucosa in AG; and finally, total replacement of foveola-type mucosa by groove-type mucosa (Fig. 8). Consequently, we suspect that the pattern of mosaic distribution and proportional change in different micro-mucosal structures and histological findings, according to the extent of CAFG, was consistent with the pathological findings of multifocal atrophic gastritis.

In previous studies that have investigated endoscopic finding of gastritis, only endoscopic images from unknown regions were related to histological findings and the area evaluated was not specified. Thus, clinical relevance is equivocal in terms of topographic variation of chronic gastritis among different gastric diseases [30]. We selected the corpus lesser curvature as a region-of-interest because this area is affected in the early phase of CAFG progression [10]. We think it is good to evaluate endoscopic finding in certain areas that are similar to the recommended biopsy sites in the updated Sydney system [18]. Then, we can relate the endoscopic findings to other studies investigating the association between histological findings and clinical manifestations. We indicated that atrophy and intestinal metaplasia in biopsy specimens taken from the corpus lesser curvature were strongly related to risk of gastric cancer, compared with biopsies from other sites of the stomach in our previous case–control study [31]. In the current study, we found a close relationship between the proportions of AG and int-AG in the corpus lesser curvature and the extent of CAFG, which was associated with the risk of development of gastric cancer. This result suggests that we could assess gastric cancer risk by the endoscopic finding in the corpus lesser curvature with IEE.

There are limitations to be considered in this study. First, we enrolled only patients with early gastric cancer, so whether this principle is applicable to other types of chronic gastritis, such as patients without gastric cancer, autoimmune gastritis, and lymphocytic gastritis, is unknown. Second, difference in the grade of histological atrophy and intestinal metaplasia between each micro-surface pattern was not as distinct as that of endoscopic image, that is, more than 60% of the foveola-type mucosa has atrophy and intestinal metaplasia. In this study, 28 patients (56%) had large extent of CAFG and 21 (42%) had int-AG-dominant (>80%) pattern that mostly showed groove-type mucosa in the region-of-interest. So an area with foveola-type mucosa was usually smaller than opening width (7 mm) of biopsy forceps in such patients. Consequently, surrounding mucosa with atrophy and intestinal metaplasia contaminated biopsy samples and it could result in the mild grade of atrophy and intestinal metaplasia of the foveola-type mucosa. Development of precise method to relate magnifying endoscopic image to histological finding is expected.

In conclusion, we clarified the patterns of AG and int-AG in relation to the extent of CAFG and their micro-mucosal structures and histological findings of chronic gastritis in the gastric corpus. The results could improve the diagnostic yield of IEE to investigate pathogenesis of CAFG.
